# Anti-Ro52 Autoantibodies Are Related to Chronic Graft-vs.-Host Disease After Allogeneic Hematopoietic Stem Cell Transplantation

**DOI:** 10.3389/fimmu.2020.01505

**Published:** 2020-07-28

**Authors:** Kaibo Yang, Yanqiu Chen, Hanzhou Qi, Yiling Ye, Zhiping Fan, Fen Huang, Haiyan Zhang, Yuan Suo, Qifa Liu, Hua Jin

**Affiliations:** ^1^Department of Hematology, Nanfang Hospital, Southern Medical University, Guangzhou, China; ^2^Guangdong Provincial Key Laboratory of Construction and Detection in Tissue Engineering, Southern Medical University, Guangdong, China

**Keywords:** chronic graft-vs.-host disease, anti-Ro52 autoantibodies, anti-nuclear autoantibodies, allogeneic hematopoietic stem cell transplantation, B-cell activating factor (BAFF)

## Abstract

Chronic graft-vs.-host disease (cGVHD) remains a major cause of morbidity and mortality after allogeneic hematopoietic stem cell transplantation (allo-HSCT). Previous studies have shown that autoantibodies play an important role in the development of cGVHD. Anti-nuclear autoantibodies (ANA) is the most frequently detected autoantibodies in patients with cGVHD, but the role of anti-Ro52 autoantibodies (anti-Ro52) in cGVHD remains largely unknown. In this study, we analyzed autoantibodies from 84 patients after allo-HSCT, including 42 with active cGVHD and 42 without cGVHD. Autoantibodies were found in 36 (42.9%) patients. Among these autoantibody-positive patients, 28 (77.8%) patients had active cGVHD. The most frequent autoantibodies in patients with active cGVHD were ANA (50.0%), anti-Ro52 (28.6%) and anti-mitochondrial autoantibodies type 2 (4.8%). We further explored the association between anti-Ro52 and cGVHD. Patients with active cGVHD had higher anti-Ro52 levels than patients without cGVHD (*P* < 0.05). The increases of anti-Ro52 levels were more significant in patients with moderate/severe cGVHD compared to those of patients without cGVHD (*P* < 0.05). Stratified and multivariable logistic regression analysis demonstrated that moderate/severe cGVHD was an independent risk factor for the levels of anti-Ro52 (*P* < 0.01). ROC analysis confirmed anti-Ro52 as a risk factor for progression of skin cGVHD. Moreover, the anti-Ro52 levels were highly correlated with the levels of B cell-activating factor (BAFF) and IgG1 antibodies. Our study demonstrates that anti-Ro52 is associated with cGVHD. The increased levels of anti-Ro52 were associated with higher levels of BAFF and IgG1 antibodies, suggesting a mechanistic link between elevated anti-Ro52 levels and aberrant B cell homeostasis.

## Introduction

Allogeneic hematopoietic stem cell transplantation (allo-HSCT) is a curative therapy for various hematological malignancies. Chronic graft-vs.-host disease (cGVHD) is a leading cause of nonrelapse mortality after allo-HSCT (1–5). The clinical symptoms of cGVHD are highly variable, including skin sclerosis, bronchiolitis obliterans, salivary, and lacrimal gland pathology (6, 7). Chronic GVHD is an autoimmune-like syndrome caused by the interactions of donor CD4^+^ T and B cells and production of IgG (7–11). Recently, antibodies have been reported to play an important role in the development of cGVHD (12–19). Previous studies showed that donor B cell-derived antibodies augmented the development of bronchiolitis obliterans and perpetuated cutaneous cGVHD in mice (7, 9). In humans, stimulatory autoantibodies against platelet-derived growth factor receptor (PDGFR), alloantibodies to Y chromosome-encoded proteins and anti-nuclear autoantibodies correlated significantly with clinical cGVHD development (20–23). Autoantibodies against the Ro52 protein (anti-Ro52 autoantibodies, anti-Ro52) can be detected in patients with autoimmune diseases such as systemic lupus erythematosus (SLE), systemic sclerosis, and Sjogren's syndrome (24). However, it is rarely reported whether anti-Ro52 can affect cGVHD in patients undergoing allo-HSCT. The purpose of this study was to explore the association between anti-Ro52 and human cGVHD.

## Materials and Methods

### Study Design and Patient Eligibility

Patients with hematological malignancy undergoing allo-HSCT were enrolled in this study. This study included 42 patients with active cGVHD. Eligibility criteria were as follows: (1) >3 months from time of allo-HSCT; (2) not received prednisone (≥0.5 mg/kg per day) 2 weeks before sample collection; and (3) never received rituximab (anti-CD20 mAb) or ibrutinib (inhibitor of Bruton's tyrosine kinase). Forty-two patients without cGVHD were matched to 42 patients with active cGVHD according to age, gender, primary disease, time after transplantation, conditioning regimen, HLA typing, source of graft, and grade of acute GVHD. This study was performed in accordance with the Declaration of Helsinki and was approved by the institutional review board of Nanfang Hospital. All patients and donors gave written informed consent to participate in the study.

### GVHD Prophylaxis and Treatment

Generally, all HLA-haploidentical donor (HID) patients were transplanted with a combination of bone marrow (BM) and peripheral blood stem cell (PBSC) grafts, whereas most HLA-matched sibling donor (MSD) patients received PBSC grafts (25, 26). Cyclosporine A (CsA), methotrexate (MTX), and mycophenolate mofetil (MMF) were administered to most patients undergoing MSD transplant for GVHD prophylaxis. CsA + MTX + MMF + antithymocyte globulin (ATG) was administered to patients undergoing HID transplants for GVHD prophylaxis (25–27). Patients received CsA, MMF and steroids for acute GVHD treatment as detailed in a previous report (28). Anti-CD25 monoclonal antibody and other immunosuppressive drugs were used to treat steroid-resistant acute GVHD. Steroids and CsA were used initially to treat cGVHD and were used in combination with various immunosuppressive agents to treat cGVHD that was unresponsive to initial therapy (29).

### GVHD Assessment

The diagnosis and grade of cGVHD on the day of sample collection, not at first diagnosis, were documented by clinical examination and laboratory testing [according to the National Institutes of Health (NIH) criteria] (30). Patients with active cGVHD were defined as requiring the addition of high-dose prednisone (≥2 mg/kg per day) or continued multiagent immunosuppression after sample collection (11, 31). Patients without cGVHD were defined as patients who had not developed cGVHD by the time of sample collection. Patients with previous cGVHD that had resolved or who became asymptomatic by the time of sample collection were not included (11, 31).

### Detection of Serum Autoantibodies

The enrolled patients were screened for the presence of the following autoantibodies: anti-Ro52 autoantibodies (anti-Ro52), anti-nuclear autoantibodies (ANA), anti-histone autoantibodies (AHA), anti-ribosomal P protein autoantibodies (anti-Rib-P), anti-polymyositis/scleroderma autoantibodies (anti-PM/Scl), anti-histidyl tRNA synthetase autoantibodies (anti-Jo-1), anti-mitochondrial autoantibodies type 2 (AMA-M2), and anti-centromere-B autoantibodies (anti-CENP-B) (Euroimmun, Lubeck, Germany). The detection of ANA was performed by indirect immunofluorescence assay (IFA) using HEp-2 cells (AESKU ANA-IFA reagent kit). Patient' s serum was diluted 1:80 and allocated into the appropriate cells and was incubated slides 30 min. After the incubation, rinsed off the serum with washing buffer in a slide staining dish and following covered with FITC labeled anti-human IgG for 30 min. Slides were washed with washing buffer and sealed with mounting medium for automatic interpretation by the HELIOS system (AESKU Diagnostics GmbH & Co. KG, Germany). The AESKU ANA-IFA reagent kit and the fully automated HELIOS system are from AESKU.DIOGNOSTICS GmbH & Co. KG. HELIOS is a system which automatically takes over the complete pipetting and image capturing of IFA tests without manual interference (32). An ANA titer of 1:80 or greater was considered positive. Patient serum samples meeting the cutoff titer of 1:80 were serially diluted to 1:640. The results were evaluated by the use of software (Euroimmun, Lubeck, Germany) and expressed in arbitrary units (AU/mL).

### Enzyme-Linked Immunosorbent Assay

The levels of soluble B cell-activating factor (BAFF) and IgG1 in patient plasma samples were measured by commercially available enzyme-linked immunosorbent assay (ELISA) (DBLYS0B R&D Systems, Minneapolis, USA and 88-50560-22, Invitrogen, CA, USA, respectively). The plates were read using the CLARIO star system following the manufacturer's recommended procedures (BMG Labtech, Cary, NC, USA).

### Statistical Analysis

The descriptive analysis of patient characteristics included median, minimum and maximum values for continuous variables and numbers and frequencies for categorical variables. Fisher's exact test was performed in comparison of categorical variables. For continuous variables, Student's *t*-test was performed for comparisons between two groups. Univariable logistic regression analysis was performed for the factors listed in Table 1 to identify variables that were associated with the presence of autoantibodies. Factors that were significant at the 0.1 level from the univariable logistic regression were included in the multivariable logistic regression. Correlation studies were performed using Pearson's correlation test. Anti-Ro52 levels, a highly skewed variable, was transformed to logarithm with base 10 for meeting the normality assumption. Receiver operating characteristic (ROC) curves analysis and area under the curve (AUC) estimation were also performed in order to discriminate our interests and the optimum cut-off value was according to the Youden's index. All statistics were analyzed in GraphPad Software (Prism Version 6.0; GraphPad Software, San Diego, CA) or SPSS version 22.0 (SPSS, Chicago, USA). Tests for significance were 2-sided, with a significance P level of 0.05 or less.

**Table 1:** Patient characteristics.

	**Chronic GVHD**		
**Characteristics**	**No (*n* = 42)**	**Active (*n* = 42)**	***P***
Age, median (range), y	30 (16–61)	30 (17–57)	0.83
Gender, no (%)			0.35
Male	31 (73.8)	26 (61.9)	
Female	11 (26.2)	16 (38.1)	
Primary disease, no (%)^a^			0.17
ALL	15 (35.7)	23 (54.7)	
AML	24 (57.1)	18 (42.9)	
Others	3 (7.2)	1 (2.4)	
Duration time from HSCT to sample collection, median (range), m	8.7 (3.1–21.1)	8.9 (3.4–19.2)	0.98
Conditioning regimen, no (%)^b^			0.59
Myeloablative	35 (83.3)	32 (76.2)	
Intensified	7 (16.7)	10 (23.8)	
HLA typing, no (%)			0.37
Matched	24 (57.1)	29 (69.0)	
Mismatched	18 (42.9)	13 (31.0)	
Source of graft, no (%)			0.12
BM + PBSC	22 (52.4)	14 (33.3)	
PBSC	20 (47.6)	28 (66.7)	
GVHD prophylaxis, no (%)^c^			<0.01
ATG based	27 (64.3)	13 (31.0)	
Non-ATG based	15 (35.7)	29 (69.0)	
Acute GVHD grade, no (%)			0.46
0–I	33 (78.6)	29 (69.0)	
II–IV	9 (21.4)	13 (31.0)	
Immunosuppressive treatments at study inclusion, no (%)^d^			<0.001
None	18 (42.9)	0 (0.0)	
1	24 (57.1)	10 (23.8)	
2	0 (0.0)	23 (54.8)	
≥ 3	0 (0.0)	9 (21.4)	
Duration of immunosuppressive medication, median (range), m	3.0 (2.0–7.0)	9.0 (3.0–18.0)	<0.01

*GVHD, graft-vs.-host disease; ALL, acute lymphoblastic leukemia; AML, acute myeloid leukemia; HSCT, hematopoietic stem cell transplantation; HLA, human leukocyte antigen; BM, bone marrow; PBSC, peripheral blood stem cell; ATG, antithymocyte globulin*.

a *The other category included aplastic anemia, myelodysplastic syndrome, and lymphoma*.

b *Myeloablative conditioning regimens include TBI (total body irradiation) + Cy (cyclophosphamide), Bu (busulfan)+ Cy, and Bu + Flu (fludarabine). Intensified conditioning regimens include TBI + Cy + etoposide, and Flu + cytarabine + TBI + Cy*.

c *Non-ATG based GVHD prophylaxis include cyclosporine A (CsA), methotrexate (MTX), and mycophenolate mofetil (MMF). ATG based GVHD prophylaxis include CsA + MTX + MMF + ATG*.

d *Immunosuppressive treatments include CsA, tacrolimus (Tac), MMF, and steroids*.

## Results

### Patient Characteristics

There were 84 patients enrolled in this study between March 2016 and March 2018. The patients had a median age of 30 years (range 16–61 years), with 57 males and 27 females. Forty-two patients had active cGVHD at the time of sample collection. The median time from onset of cGVHD to the sample collection was 1.0 month (range 0.0–15.2 months). The median time from onset of immunosuppressive medication to the sample collection was 5.0 months (range 2.0–18.0 months). There were no significant differences in age, gender, primary disease, time after transplantation, conditioning regimen, HLA typing, source of graft, and grade of acute GVHD between patients with and without cGVHD in our study (Table 1). Of the 42 patients with active cGVHD, 11 patients had mild cGVHD, 21 patients had moderate cGVHD, and 10 patients had severe cGVHD. The most frequent organ manifestations of cGVHD were skin (50.0%) and oral mucosa (28.6%). Twelve patients (28.6%) had more than two organs involved (Table 2). At a median follow-up of 8.4 months (range 3.1–17.2 months) post-transplantation, two of 42 patients without cGVHD subsequently developed cGVHD 3.5 and 8.9 months later.

**Table 2:** Clinical characteristics of cGVHD.

**Organ**	**Mild**	**Moderate**	**Severe**
	***N* = 11**	***N* = 21**	***N* = 10**
Skin (%)	3 (7.2)	12 (28.6)	6 (14.2)
Eyes (%)	0 (0.0)	0 (0.0)	2 (4.8)
Oral mucosa (%)	6 (14.2)	3 (7.2)	3 (7.2)
Liver (%)	0 (0.0)	7 (16.7)	3 (7.2)
Gastrointestinal (%)	1 (2.4)	1 (2.4)	0 (0.0)
Lungs (%)	0 (0.0)	2 (4.8)	4 (9.5)
Joints (%)	1 (2.4)	2 (4.8)	1 (2.4)
Genital tract (%)	0 (0.0)	0 (0.0)	1 (2.4)
Mean prednisone-equivalent	0 (0.0)	9.3 (0.0–20.0)	14.5 (5.0–25.0)
steroid dose (range), mg			
Duration of cGVHD until sampling,	0 (0.0)	3.0 (0.0–12.0)	5.5 (0.6–15.2)
median (range), m			

### Prevalence of Autoantibodies

**Figure 1:**
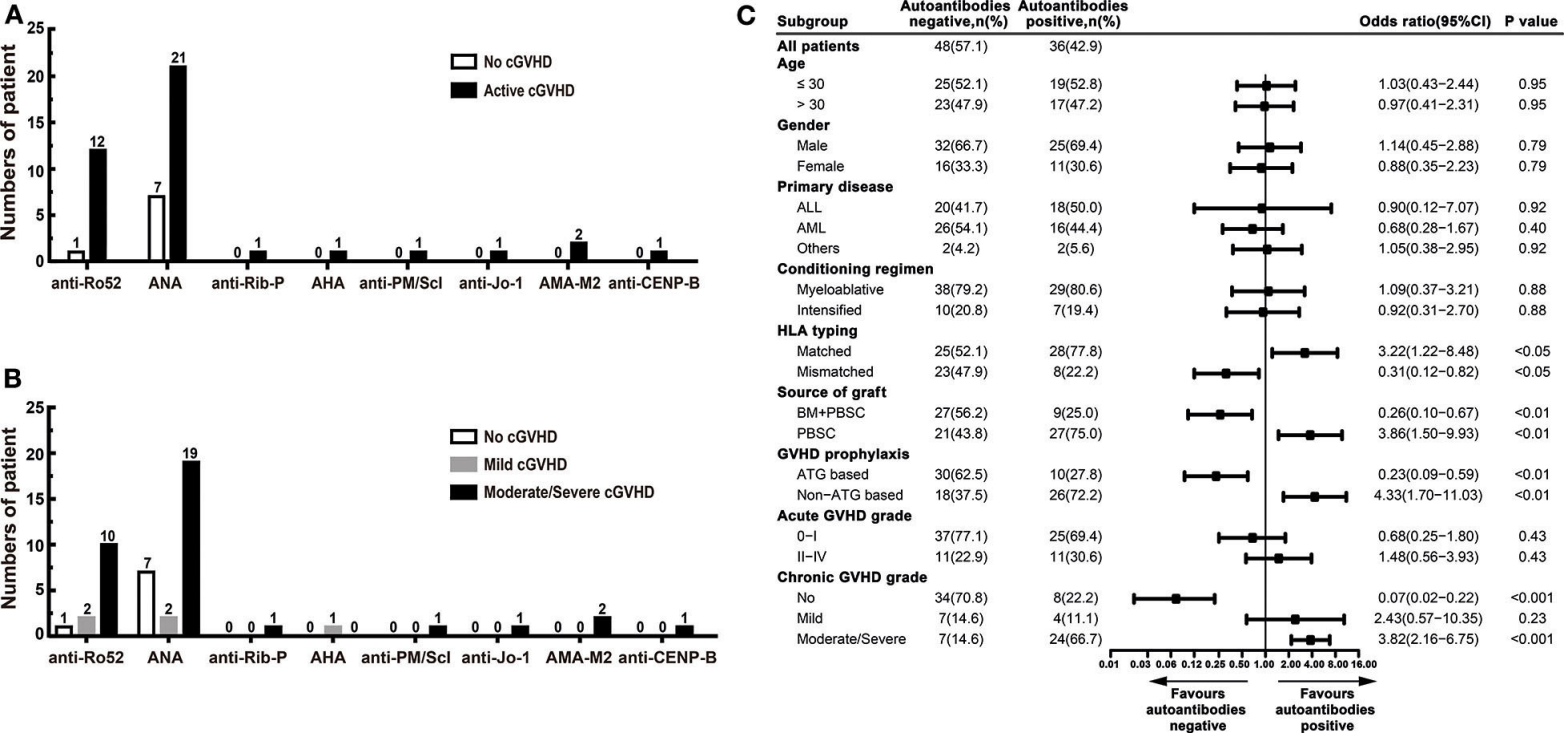
The prevalence of autoantibodies in patients after allo-HSCT. **(A)** The numbers of positive autoantibodies in patients without cGVHD and patients with active cGVHD. **(B)** The numbers of positive autoantibodies in patients with different severities of cGVHD. **(C)** Stratified analysis for factors associated with the presence of autoantibodies. The black bars in the forest plot indicate odds ratios with 95% confidence intervals for each variable. cGVHD, chronic graft-vs.-host disease; anti-Ro52, anti-Ro52 autoantibodies; ANA, anti-nuclear autoantibodies; anti-Rib-P, anti-ribosomal P protein autoantibodies; AHA, anti-histone autoantibodies; anti-PM/Scl, anti-polymyositis/scleroderma autoantibodies; anti-Jo-1, anti-histidyl tRNA synthetase autoantibodies; AMAM-2, anti-mitochondrial autoantibodies type 2; anti-CENP-B, anti-centromere-B autoantibodies; 95% CI, 95% confidence interval; ALL, acute lymphoblastic leukemia; AML, acute myeloid leukemia; HLA, human leukocyte antigen; BM, bone marrow; PBSC, peripheral blood stem cell; ATG, antithymocyte globulin.

Autoantibodies were detected in 36 (42.9%) patients, including 28 (77.8%) patients had active cGVHD, and 8 (22.2%) patients had no cGVHD. Autoantibodies were not found in 48 (57.1%) patients: 34 (70.8%) patients had no cGVHD, and 14 (29.2%) patients had active cGVHD. Ten patients had two or more autoantibodies. The most frequent autoantibodies in patients with active cGVHD were ANA and anti-Ro52. ANA were found in 21 (50.0%) active cGVHD patients: anti-Ro52 in 12 (28.6%), anti-Rib-P in 1 (2.4%), AHA in 1 (2.4%), anti-PM/Scl in 1 (2.4%), anti-Jo-1 in 1 (2.4%), AMA-M2 in 2 (4.8%), and anti-CENP-B in 1 (2.4%) (Figure 1A). Patients with moderate/severe cGVHD had a higher proportion of autoantibody positivity than patients with mild cGVHD, especially ANA and anti-Ro52 (Table 3). The proportion of patients with ANA positivity was 19/21 (90.5%) in patients with moderate/severe cGVHD and 2/21 (9.5%) in patients with mild cGVHD. The proportion of patients with anti-Ro52 positivity was 10/12 (83.3%) in patients with moderate/severe cGVHD and 2/12 (16.7%) in patients with mild cGVHD (Figure 1B).

**Table 3:** Comparison of autoantibodies among patients with different cGVHD grade.

	**Chronic GVHD grade**						
	**No** **(*****n*** **=** **42)**		**Mild** **(*****n*** **=** **11)**		**Moderate/Severe** **(*****n*** **=** **31)**		***P***
**Autoantibodies**	**Negative**	**Positive**	**Negative**	**Positive**	**Negative**	**Positive**	
Anti-Ro52	41 (97.6)	1 (2.4)	9 (81.8)	2 (18.2)	21 (67.7)	10 (32.3)	<0.01
ANA	35 (83.3)	7 (16.7)	9 (81.8)	2 (18.2)	12 (38.7)	19 (61.3)	<0.001
Anti-Rib-P	42 (100.0)	0 (0.0)	11 (100.0)	0 (0.0)	30 (96.8)	1 (3.2)	0.42
AHA	42 (100.0)	0 (0.0)	10 (90.9)	1 (9.1)	31 (100.0)	0 (0.0)	<0.05
Anti-PM/Scl	42 (100.0)	0 (0.0)	11 (100.0)	0 (0.0)	30 (96.8)	1 (3.2)	0.42
Anti-Jo-1	42 (100.0)	0 (0.0)	11 (100.0)	0 (0.0)	30 (96.8)	1 (3.2)	0.42
AMA-M2	42 (100.0)	0 (0.0)	11 (100.0)	0 (0.0)	29 (93.5)	2 (6.5)	0.17
Anti-CENP-B	42 (100.0)	0 (0.0)	11 (100.0)	0 (0.0)	30 (96.8)	1 (3.2)	0.42

*GVHD, graft-vs.-host disease; anti-Ro52, anti-Ro52 autoantibodies; ANA, anti-nuclear autoantibodies; anti-Rib-P, anti-ribosomal P protein autoantibodies; AHA, anti-histone autoantibodies; anti-PM/Scl, anti-polymyositis/scleroderma autoantibodies; anti-Jo-1, anti-histidyl tRNA synthetase autoantibodies; AMA-M2, anti-mitochondrial autoantibodies type 2; anti-CENP-B, anti-centromere-B autoantibodies*.

### Association Between Autoantibodies and cGVHD

There were no statistically significant differences in age, gender, primary disease, conditioning regimen, and acute GVHD grade between patients who developed autoantibodies and patients who did not develop autoantibodies. Compared with patients who did not develop autoantibodies, patients who developed autoantibodies have several characteristics, including HLA-matched transplant, PBSC graft, non-ATG based GVHD prophylaxis, and moderate/severe cGVHD (Table 4). Further stratified and multivariable logistic regression analysis demonstrated that moderate/severe cGVHD was an independent risk factor for the levels of autoantibodies (*P* < 0.001) (Figure 1C and Table 5).

**Table 4:** Comparison of clinical characteristics between patients who developed autoantibodies and patients who did not develop autoantibodies.

	**Autoantibodies**		
**Characteristics**	**Negative** **(*n* = 48)**	**Positive** **(*n* = 36)**	***P***
Age, median (range), y	30 (16–61)	29 (17–51)	1.00
Gender, no (%)			0.82
Male	32 (66.7)	25 (69.4)	
Female	16 (33.3)	11 (30.6)	
Primary disease, no (%)^a^			0.67
ALL	20 (41.7)	18 (50.0)	
AML	26 (54.1)	16 (44.4)	
Others	2 (4.2)	2 (5.6)	
Conditioning regimen, no (%)^b^			1.00
Myeloablative	38 (79.2)	29 (80.6)	
Intensified	10 (20.8)	7 (19.4)	
HLA typing, no (%)			<0.05
Matched	25 (52.1)	28 (77.8)	
Mismatched	23 (47.9)	8 (22.2)	
Source of graft, no (%)			<0.01
BM + PBSC	27 (56.2)	9 (25.0)	
PBSC	21 (43.8)	27 (75.0)	
GVHD prophylaxis, no (%)^c^			<0.01
ATG based	30 (62.5)	10 (27.8)	
Non-ATG based	18 (37.5)	26 (72.2)	
Acute GVHD grade, no (%)			0.46
0-I	37 (77.1)	25 (69.4)	
II-IV	11 (22.9)	11 (30.6)	
Chronic GVHD grade, no (%)			<0.001
No	34 (70.8)	8 (22.2)	
Mild	7 (14.6)	4 (11.1)	
Moderate/Severe	7 (14.6)	24 (66.7)	

*ALL, acute lymphoblastic leukemia; AML, acute myeloid leukemia; HLA, human leukocyte antigen; BM, bone marrow; PBSC, peripheral blood stem cell; GVHD, graft-vs.-host disease; ATG, antithymocyte globulin*.

a *The other category included aplastic anemia, myelodysplastic syndrome, and lymphoma*.

b *Myeloablative conditioning regimens include TBI (total body irradiation) + Cy (cyclophosphamide), Bu (busulfan)+ Cy, and Bu + Flu (fludarabine). Intensified conditioning regimens include TBI + Cy + etoposide, and Flu + cytarabine + TBI + Cy*.

c *Non-ATG based GVHD prophylaxis include cyclosporine A (CsA), methotrexate (MTX), and mycophenolate mofetil (MMF). ATG based GVHD prophylaxis include CsA + MTX + MMF + ATG*.

**Table 5:** Logistic regression for factors associated with the levels of autoantibodies.

**Characteristics**	**Contrast**	**Univariable**			**Multivariable**		
		**OR estimate**	**95% CI**	***P***	**OR estimate**	**95% CI**	***P***
Age	≤30 vs. >30	0.97	0.41–2.31	0.95			
Gender	Male vs. Female	0.88	0.35–2.23	0.79			
Primary disease^a^	ALL vs. AML vs. Others	0.81	0.38–1.72	0.59			
Conditioning regimen^b^	Myeloablative vs. Intensified	0.92	0.31–2.70	0.88			
HLA typing	Matched vs. Mismatched	0.31	0.12–0.82	<0.05	0.63	0.04–11.30	0.76
Source of graft	BM+PBSC vs. PBSC	3.86	1.50–9.93	<0.01	1.99	0.18–21.95	0.57
GVHD prophylaxis^c^	ATG based vs. Non-ATG based	4.33	1.70–11.03	<0.01	1.14	0.18–7.32	0.89
Acute GVHD grade	0–I vs. II–IV	1.48	0.56–3.93	0.43			
Chronic GVHD grade	No vs. Mild vs. Moderate/Severe	3.80	2.15–6.71	<0.001	3.65	1.93–6.92	<0.001

*OR, odds ratio; 95% CI, 95% confidence interval; ALL, acute lymphoblastic leukemia; AML, acute myeloid leukemia; HLA, human leukocyte antigen; BM, bone marrow; PBSC, peripheral blood stem cell; GVHD, graft-vs.-host disease; ATG, antithymocyte globulin*.

a *The other category included aplastic anemia, myelodysplastic syndrome, and lymphoma*.

b *Myeloablative conditioning regimens include TBI (total body irradiation) + Cy (cyclophosphamide), Bu (busulfan)+ Cy, and Bu + Flu (fludarabine). Intensified conditioning regimens include TBI + Cy + etoposide, and Flu + cytarabine + TBI + Cy*.

c *Non-ATG based GVHD prophylaxis include cyclosporine A (CsA), methotrexate (MTX), and mycophenolate mofetil (MMF). ATG based GVHD prophylaxis include CsA + MTX + MMF + ATG*.

**Figure 2:**
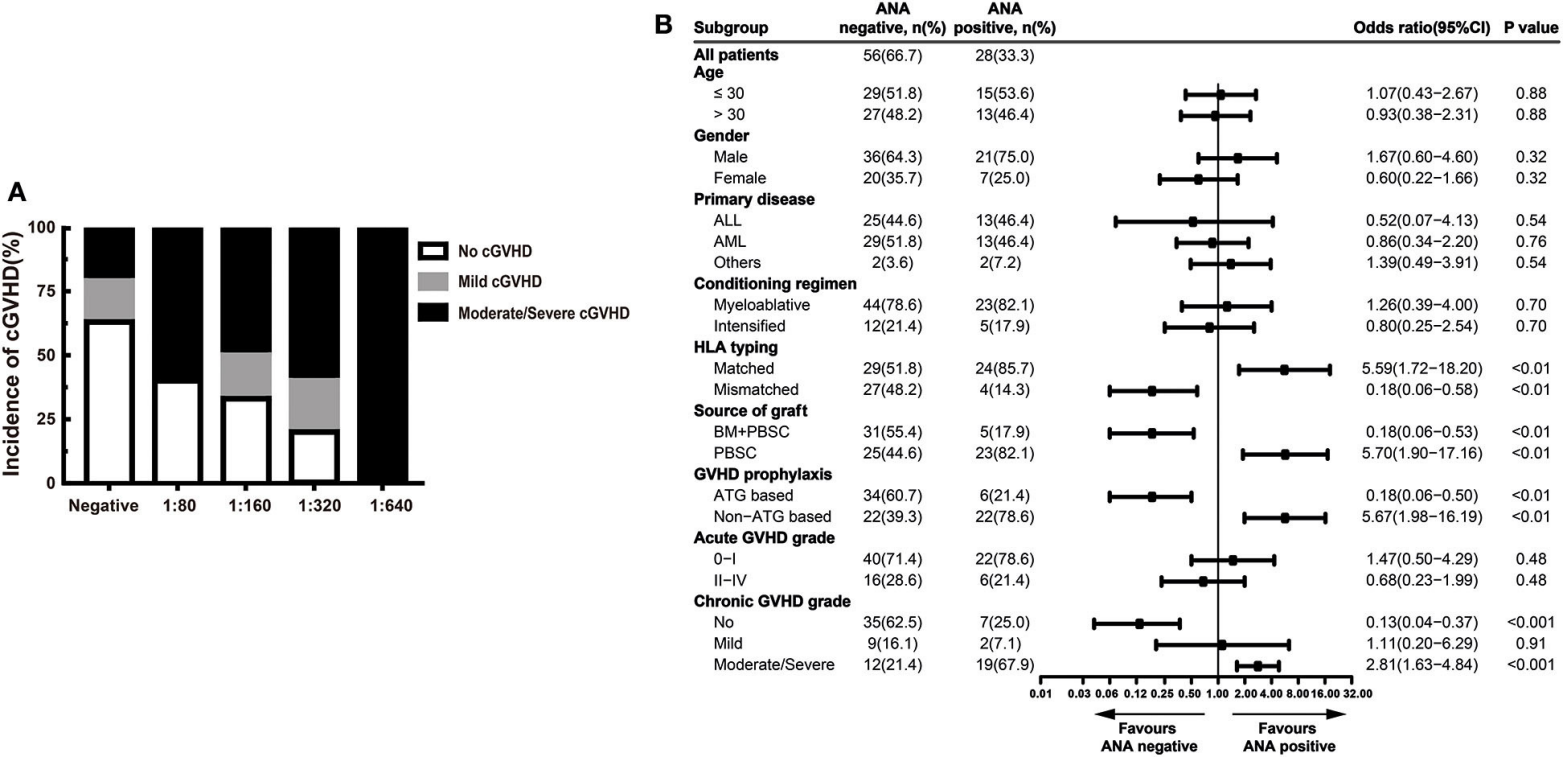
ANA increased in patients with active cGVHD. **(A)** The proportion of patients with different severities of cGVHD according to different ANA titers. **(B)** Stratified analysis for factors associated with the presence of ANA. The black bars in the forest plot indicate odds ratios with 95% confidence intervals for each variable. cGVHD, chronic graft-vs.-host disease; ANA, anti-nuclear autoantibodies; 95% CI, 95% confidence interval; ALL, acute lymphoblastic leukemia; AML, acute myeloid leukemia; HLA, human leukocyte antigen; BM, bone marrow; PBSC, peripheral blood stem cell; ATG, antithymocyte globulin.

In our study, higher ANA prevalence was also detected in patients with active cGVHD than patients without GVHD (Figure 1A). Moreover, we compared different ANA titers among patients without cGVHD, patients with mild cGVHD, and patients with moderate/severe cGVHD. Regardless of the titers, patients with moderate/severe cGVHD had higher titers than patients with mild cGVHD [1:80 (60.0%), 1:160 (50.0%), 1:320 (60.0%) and 1:640 (100.0%) vs. 1:80 (0.0%), 1:160 (17.0%), 1:320 (20.0%), and 1:640 (0.0%)] (Figure 2A). Further stratified and multivariable logistic regression analysis demonstrated that moderate/severe cGVHD was an independent risk factor for the levels of ANA (*P* < 0.01) (Figure 2B and Table 6).

**Table 6:** Logistic regression for factors associated with the levels of ANA.

**Characteristics**	**Contrast**	**Univariable**			**Multivariable**		
		**OR estimate**	**95% CI**	***P***	**OR estimate**	**95% CI**	***P***
Age	≤30 vs. >30	0.93	0.38–2.31	0.88			
Gender	Male vs. Female	0.60	0.22–1.66	0.32			
Primary disease^a^	ALL vs. AML vs. Others	1.05	0.48–2.30	0.89			
Conditioning regimen^b^	Myeloablative vs. Intensified	0.80	0.25–2.54	0.70			
HLA typing	Matched vs. Mismatched	0.18	0.06–0.58	<0.01	0.26	0.01–5.01	0.37
Source of graft	BM+PBSC vs. PBSC	5.70	1.90–17.16	<0.01	1.58	0.15–17.06	0.71
GVHD prophylaxis^c^	ATG based vs. Non-ATG based	5.67	1.98–16.19	<0.01	0.96	0.14–6.36	0.97
Acute GVHD grade	0–I vs. II–IV	0.68	0.23–1.99	0.48			
Chronic GVHD grade	No vs. Mild vs. Moderate/Severe	2.87	1.65–5.00	<0.001	2.84	1.47–5.49	<0.01

*OR, odds ratio; 95% CI, 95% confidence interval; ALL, acute lymphoblastic leukemia; AML, acute myeloid leukemia; HLA, human leukocyte antigen; BM, bone marrow; PBSC, peripheral blood stem cell; GVHD, graft-vs.-host disease; ATG, antithymocyte globulin*.

a *The other category included aplastic anemia, myelodysplastic syndrome, and lymphoma*.

b *Myeloablative conditioning regimens include TBI (total body irradiation) + Cy (cyclophosphamide), Bu (busulfan)+ Cy, and Bu + Flu (fludarabine). Intensified conditioning regimens include TBI + Cy + etoposide, and Flu + cytarabine + TBI + Cy*.

c *Non-ATG based GVHD prophylaxis include cyclosporine A (CsA), methotrexate (MTX), and mycophenolate mofetil (MMF). ATG based GVHD prophylaxis include CsA + MTX + MMF + ATG*.

### Association Between Anti-Ro52 and cGVHD

**Figure 3:**
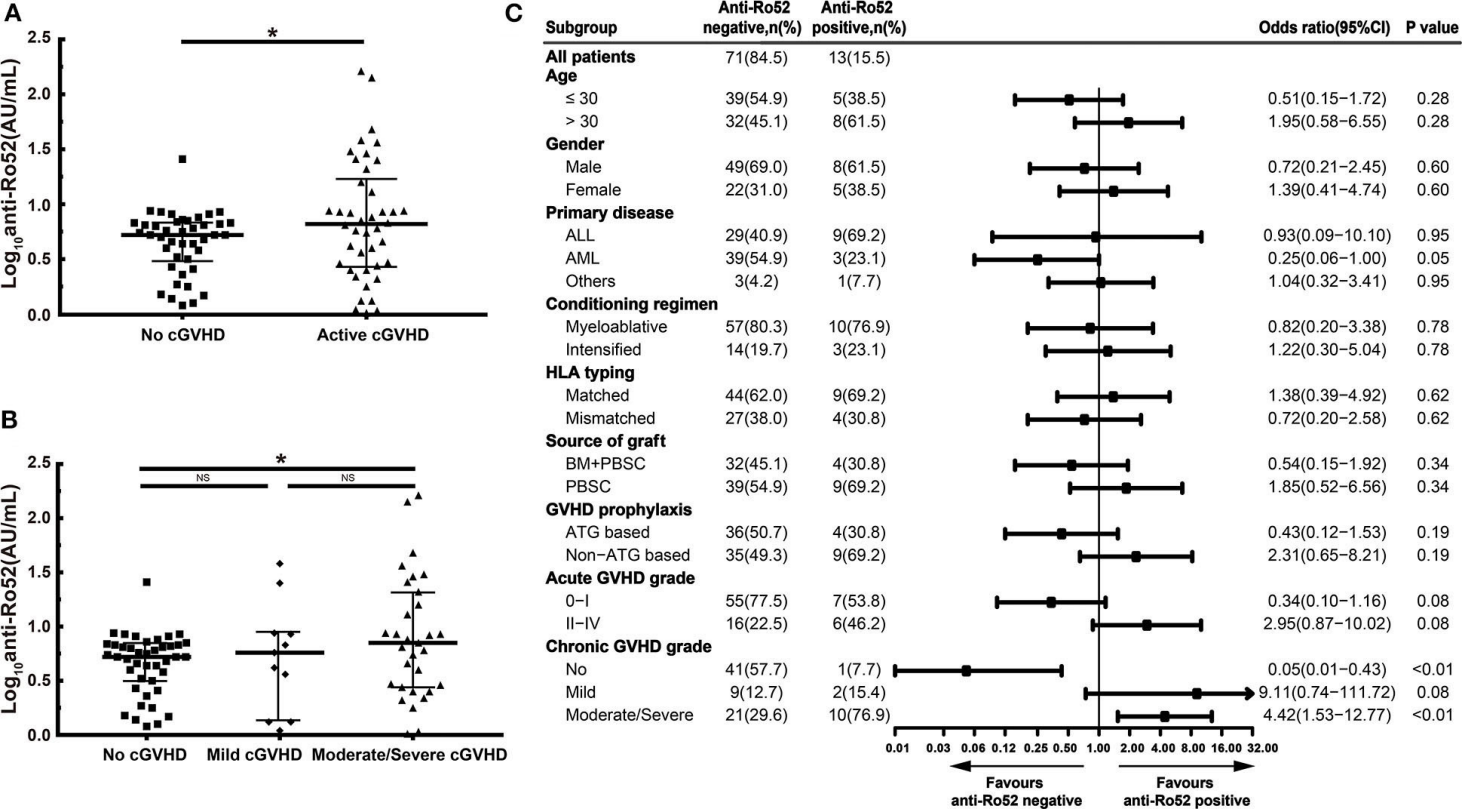
Anti-Ro52 increased in patients with active cGVHD. **(A)** Log-transformed anti-Ro52 levels in patients without cGVHD and patients with active cGVHD. **(B)** Log-transformed anti-Ro52 levels in patients with different severities of cGVHD. **(C)** Stratified analysis for factors associated with the presence of anti-Ro52 autoantibodies. The values of anti-Ro52 autoantibodies in each figure are transformed through a base-10 logarithm. The black bars in **(A,B)** represent the 75th percentile, median and 25th percentile values. The black bars in **(C)** indicate odds ratios with 95% confidence intervals for each variable. *P < 0.05. NS, not significant; anti-Ro52, anti-Ro52 autoantibodies; cGVHD, chronic graft-vs.-host disease; 95% CI, 95% confidence interval; ALL, acute lymphoblastic leukemia; AML, acute myeloid leukemia; HLA, human leukocyte antigen; BM, bone marrow; PBSC, peripheral blood stem cell; ATG, antithymocyte globulin.

In our study, patients with active cGVHD had higher anti-Ro52 levels than patients without cGVHD (*P* < 0.05) (Figure 3A). These increases of anti-Ro52 levels were more significant in patients with moderate/severe cGVHD compared to those of patients without cGVHD (median, 7.0 vs. 5.3 AU/mL; *P* < 0.05) (Figure 3B). Further stratified and multivariable logistic regression analysis demonstrated that moderate/severe cGVHD was an independent risk factor for the levels of anti-Ro52 (*P* < 0.01) (Figure 3C and Table 7).

**Table 7:** Logistic regression for factors associated with the levels of anti-Ro52.

**Characteristics**	**Contrast**	**Univariable**			**Multivariable**		
		**OR estimate**	**95% CI**	***P***	**OR estimate**	**95% CI**	***P***
Age	≤30 vs. >30	1.95	0.58–6.55	0.28			
Gender	Male vs. Female	1.39	0.41–4.74	0.60			
Primary disease^a^	ALL vs. AML vs. Others	0.45	0.14–1.38	0.16			
Conditioning regimen^b^	Myeloablative vs. Intensified	1.22	0.30–5.04	0.78			
HLA typing	Matched vs. Mismatched	0.72	0.20–2.58	0.62			
Source of graft	BM+PBSC vs. PBSC	1.85	0.52–6.56	0.34			
GVHD prophylaxis^c^	ATG based vs. Non-ATG based	2.31	0.65–8.21	0.19			
Acute GVHD grade	0–I vs. II–IV	2.95	0.87–10.02	0.08	2.37	0.62–9.01	0.21
Chronic GVHD grade	No vs. Mild vs. Moderate/Severe	3.86	1.59–9.37	<0.01	3.67	1.51–8.91	<0.01

*OR, odds ratio; 95% CI, 95% confidence interval; ALL, acute lymphoblastic leukemia; AML, acute myeloid leukemia; HLA, human leukocyte antigen; BM, bone marrow; PBSC, peripheral blood stem cell; GVHD, graft-vs.-host disease; ATG, antithymocyte globulin*.

a *The other category included aplastic anemia, myelodysplastic syndrome, and lymphoma*.

b *Myeloablative conditioning regimens include TBI (total body irradiation) + Cy (cyclophosphamide), Bu (busulfan)+ Cy, and Bu + Flu (fludarabine). Intensified conditioning regimens include TBI + Cy + etoposide, and Flu + cytarabine + TBI + Cy*.

c *Non-ATG based GVHD prophylaxis include cyclosporine A (CsA), methotrexate (MTX), and mycophenolate mofetil (MMF). ATG based GVHD prophylaxis include CsA + MTX + MMF + ATG*.

### Correlation Between Anti-Ro52 and cGVHD Target Organ

**Figure 4:**
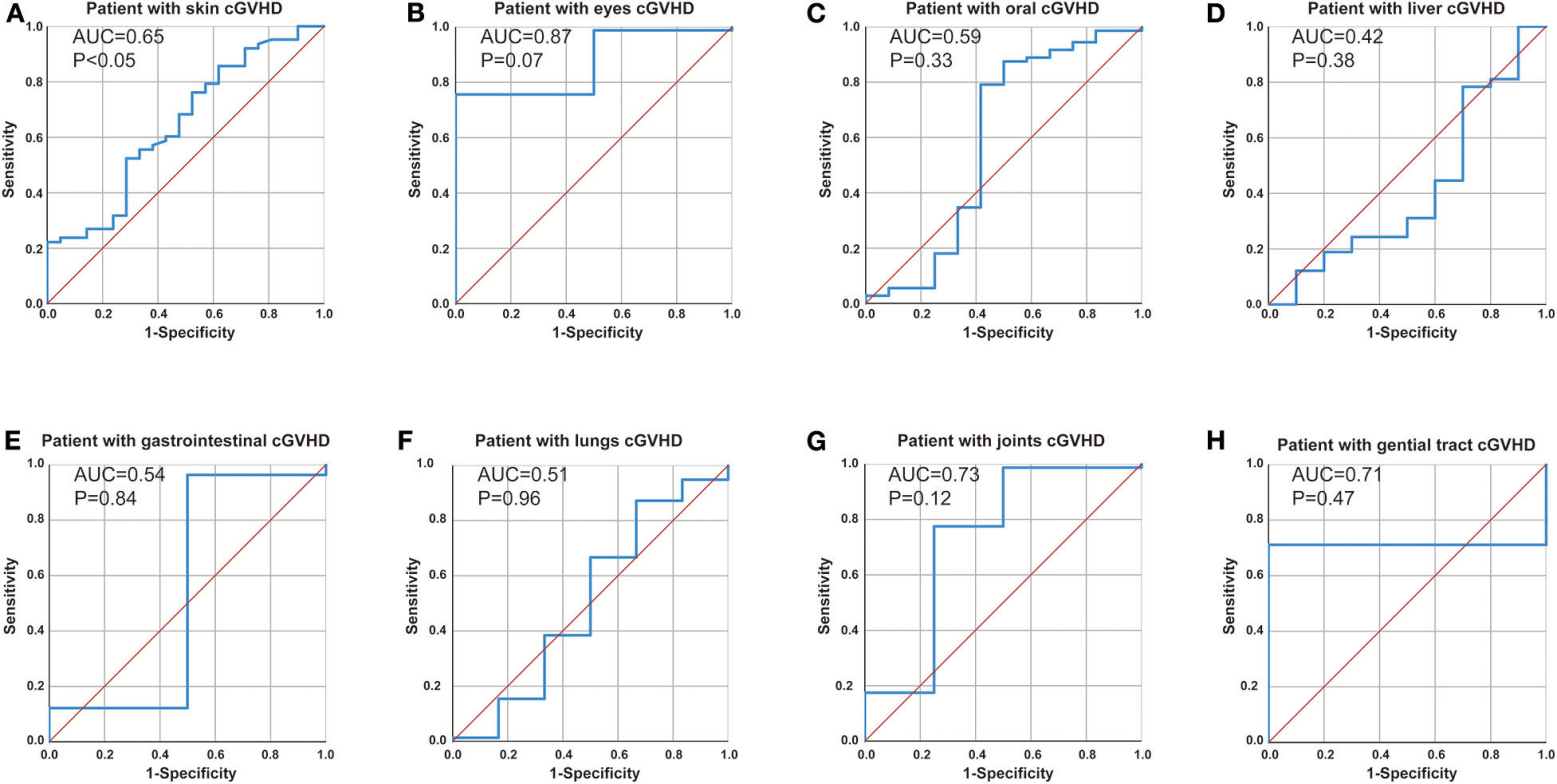
Correlation between anti-Ro52 and cGVHD target organ. Receiver operating characteristic (ROC) curve analysis to assess the association of anti-Ro52 levels in **(A)** patients with skin cGVHD vs. non-skin cGVHD, **(B)** patients with eyes cGVHD vs. non-eyes cGVHD, **(C)** patients with oral cGVHD vs. non-oral cGVHD, **(D)** patients with liver cGVHD vs. non-liver cGVHD, **(E)** patients with gastrointestinal cGVHD vs. non-gastrointestinal cGVHD, **(F)** patients with lungs cGVHD vs. non-lungs cGVHD, **(G)** patients with joints cGVHD vs. non-joints cGVHD, **(H)** patients with genital tract cGVHD vs. non-genital tract cGVHD. AUC, area under the curve. cGVHD, chronic GVHD.

We further explored the correlation between anti-Ro52 and cGVHD target organ by receiver operating characteristic (ROC) analyses. ROC analysis confirmed anti-Ro52 as a risk factor for progression of skin cGVHD (Figure 4A, cut-off = 8.60 at 85.7% sensitivity and 61.9% specificity, *P* < 0.05) but showed no correlation with other cGVHD target organs (Figures 4B–H).

### Anti-Ro52 Correlated With the Generation of B-Cell Activating Factor (BAFF) and IgG1

**Figure 5:**
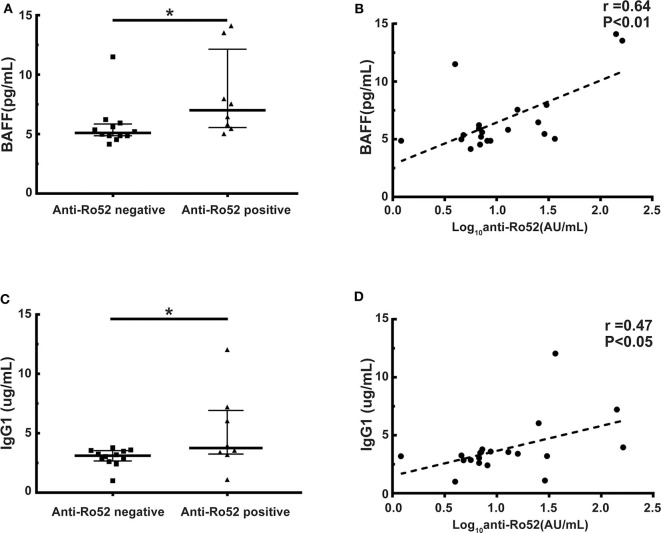
Anti-Ro52 levels are correlated with the levels of BAFF and IgG1. **(A)** BAFF levels in anti-Ro52-negative patients and anti-Ro52-positive patients. **(B)** Correlation between the levels of anti-Ro52 and the levels of BAFF in patient samples. **(C)** IgG1 levels in anti-Ro52-negative patients and anti-Ro52-positive patients. **(D)** Correlation between the levels of anti-Ro52 and the levels of IgG1 in patient samples. The black bars in each figure represent the 75th percentile, median and 25th percentile values. *P < 0.05. Anti-Ro52, anti-Ro52 autoantibodies; BAFF, B cell-activating factor.

It has been widely demonstrated that B cell homeostasis altered and BAFF and IgG1 levels increased in cGVHD patients (11, 33–35). We further examined whether anti-Ro52 was correlated with BAFF and IgG1 levels in these patients. Patients with anti-Ro52 positive had significantly higher BAFF levels than patients with anti-Ro52 negative (median, 7.0 vs. 5.1 pg/mL; *P* < 0.05) (Figure 5A). Importantly, the anti-Ro52 levels were strongly correlated with the levels of BAFF (*r* = 0.64, *P* < 0.01) (Figure 5B). A higher level of IgG1 was observed in patients with anti-Ro52 positive when compared to patients with anti-Ro52 negative (median, 3.8 vs. 3.1 μg/mL; *P* < 0.05) (Figure 5C). The levels of anti-Ro52 were also strongly correlated with the levels of IgG1 (*r* = 0.47, *P* < 0.05) (Figure 5D).

## Discussion

Recently, antibodies have been reported to play an important role in the development of cGVHD (7, 33, 36). Srinivasan et al. showed that donor B cell-derived antibodies augmented the development of bronchiolitis obliterans in a murine model of cGVHD (9). Immunoglobulin G (IgG) deposition in the skin has been observed in murine cGVHD models (7, 37). We previously reported that donor B cell antibodies augment cutaneous cGVHD in mice by damaging the thymus and increasing tissue infiltration of pathogenic Th17 cells (7). In humans, Miklos et al. reported that alloantibodies to Y chromosome-encoded proteins correlated significantly with clinical cGVHD development (21, 22). Our previous study showed that the levels of IgG1 correlated significantly with clinical cGVHD severity (11). It has also been demonstrated that circulating autoantibodies are associated with the development of clinical cGVHD (20, 23, 38). In this study, autoantibodies were detected in 36 (42.9%) patients: 28 (77.8%) patients had active cGVHD, and 8 (22.2%) patients had no cGVHD. The most common autoantibodies in patients with active cGVHD were ANA and anti-Ro52. ANA and anti-Ro52 were found in 21 (50.0%) and 12 (28.6%) active cGVHD patients, respectively. Anti-Rib-P, AHA, anti-PM/Scl, anti-Jo-1, AMA-M2, and anti-CENP-B were detected in 2.4–4.8% of cGVHD patients. Patriarca et al. found a significant association between the occurrence of ANA and cGVHD development (23), which is consistent with our findings. In our study, patients with moderate/severe cGVHD had a trend toward higher ANA titers than patients without cGVHD (≥1:160: 41.9 vs. 7.1%, *P* < 0.01). Among 42 patients without cGVHD, two patients subsequently developed cGVHD 3.5 and 8.9 months later. These results indicate that autoantibodies are not initiated but augmented the development of cGVHD. These findings are consistent with our previous findings that antibodies from donor B cells perpetuate cutaneous cGVHD in mice (7).

Ro52 is a RING finger protein that belongs to the tripartite motif family (TRIM) (24, 39). Ro52 was identified as a major autoantigen in autoimmune disease, including rheumatoid arthritis, SLE, and Sjögren's syndrome (40–42). Like several other TRIM proteins, Ro52 acts in the process of ubiquitination and regulates immune responses by targeting key molecules involved in cell proliferation, survival or death (43–45). Several studies demonstrated that increased expression of the Ro52 autoantigen might be directly involved in the reduced cellular proliferation and increased apoptotic cell death observed in Sjögren's syndrome and SLE patients and might contribute to the autoantigenic load and induction of autoimmune B and T cell responses observed in rheumatic patients (45, 46). Therefore, anti-Ro52 can be detected in patients with several different autoimmune diseases (47–49). In SLE as well as systemic sclerosis and autoimmune myositis patients, anti-Ro52 is detected in approximately one-third of the patients (50, 51). Anti-Ro52 is also the most common specificity in patients with primary Sjögren's syndrome (66.7%) (52). The presence of anti-Ro52, either as a single specificity or in a combination with other specificities, is a factor associated with interstitial lung disease (53, 54). However, the presence of anti-Ro52 in the cGVHD patients is rarely reported (55, 56). Sarantopoulos et al. reported that the levels of anti-Ro52 in patients with unresponsive cGVHD after rituximab treatment increased (56). In our study, we found that patients with active cGVHD had higher anti-Ro52 levels than patients without cGVHD (*P* < 0.05). These increases of anti-Ro52 levels were more significant in patients with moderate/severe cGVHD compared to those of patients without cGVHD (median, 7.0 vs. 5.3 AU/mL; *P* < 0.05). Further stratified and multivariable logistic regression analysis demonstrated that moderate/severe cGVHD was an independent risk factor for the levels of anti-Ro52 (*P* < 0.01). ROC analysis confirmed anti-Ro52 as a risk factor for progression of skin cGVHD.

The presence of autoantibodies emphasizes the importance of B cells in the development of cGVHD (7, 9, 23, 33, 36–38). The important role of B cells has also been confirmed by the successful treatment of some subgroups of cGVHD patients with the B cell-depleting agent rituximab (57–60). It has been reported that Ro52 can bind to almost all B cells due to its interaction with the Fc domain of IgM and IgG. By binding directly to the B cell receptor, Ro52 might be capable of activating B cells in the absence of conventional immune receptor interactions (61, 62). It has been widely demonstrated that B cell homeostasis altered and BAFF increased in cGVHD patients (33–35). BAFF expression might be indirectly regulated by Ro52 (63, 64). We further examined whether anti-Ro52 was correlated with the levels of BAFF in these patients. Patients with anti-Ro52 positive had significantly higher BAFF levels than patients with anti-Ro52 negative (median, 7.0 vs. 5.1 pg/mL; *P* < 0.05). Importantly, the levels of anti-Ro52 were strongly correlated with the levels of BAFF (*r* = 0.64, *P* < 0.01). Several investigators have demonstrated that Ro52 might bind the Fc part of IgG molecules via the B30.2/PRYSPRY domain with unexpectedly high affinity. Ro52 functionally regulates quality control of IgG1 in B cells or plasma cells through the endoplasmic reticulum-associated degradation (ERAD) system (65–67). It has also been reported that the levels of IgG, especially IgG1, increased in Ro52-null mice with dermatitis (68). Our previous study showed that the levels of IgG1 correlated significantly with clinical cGVHD severity (11). We further examined the correlation between anti-Ro52 and IgG1 levels. A higher level of IgG1 was observed in patients with anti-Ro52 positive when compared to patients with anti-Ro52 negative (median, 3.8 vs. 3.1 μg/mL; *P* < 0.05). The levels of anti-Ro52 were also strongly correlated with the levels of IgG1 (*r* = 0.47, *P* < 0.05). Espinosa et al. observed that loss of the lupus autoantigen Ro52 induced tissue inflammation and systemic autoimmunity by dysregulating the IL-23-Th17 pathway (68). The development of cGVHD is mediated by pathogenic Th17 cells (7, 69). Further studies are needed to explore whether anti-Ro52 are associated with Th17 cell development in cGVHD patients.

One limitation of this study was the limited sample size of patients. A kinetic study of anti-Ro52 prevalence was absent. Kinetic studies of more patients will be conducted to explore the effect of anti-Ro52 on cGVHD development.

## Conclusion

Our study demonstrates that the anti-Ro52 is associated with cGVHD. ROC analysis confirmed anti-Ro52 as a risk factor for progression of skin cGVHD. The levels of anti-Ro52 correlated with the severity of cGVHD and the levels of BAFF and IgG1 antibodies. Therefore, our findings support a mechanistic link between elevated anti-Ro52 levels and aberrant B cell homeostasis. Further studies will be needed to investigate the exact mechanisms of anti-Ro52 in cGVHD.

## Data Availability Statement

The raw data supporting the conclusions of this article will be made available by the authors, without undue reservation. Requests to access the datasets should be directed to Hua Jin, echohua1124@163.com.

## Ethics Statement

The studies involving human participants were reviewed and approved by Nanfang Hospital. The patients/participants provided their written informed consent to participate in this study.

## Author Contributions

KY analyzed the data and wrote the manuscript. YC and HQ collected and analyzed the data. YY, ZF, FH, HZ, and YS assisted in the research. HJ and QL designed the study, supervised the research, and critically revised the manuscript. All authors contributed to the article and approved the submitted version.

## Conflict of Interest

The authors declare that the research was conducted in the absence of any commercial or financial relationships that could be construed as a potential conflict of interest.
